# Evidence for Hitchhiking of Deleterious Mutations within the Human Genome

**DOI:** 10.1371/journal.pgen.1002240

**Published:** 2011-08-25

**Authors:** Sung Chun, Justin C. Fay

**Affiliations:** 1 Computational and Systems Biology Program, Washington University, St. Louis, Missouri, United States of America; 2 Department of Genetics and Center for Genome Sciences and Systems Biology, Washington University, St. Louis, Missouri, United States of America; University of Chicago, Howard Hughes Medical Institute, United States of America

## Abstract

Deleterious mutations present a significant obstacle to adaptive evolution. Deleterious mutations can inhibit the spread of linked adaptive mutations through a population; conversely, adaptive substitutions can increase the frequency of linked deleterious mutations and even result in their fixation. To assess the impact of adaptive mutations on linked deleterious mutations, we examined the distribution of deleterious and neutral amino acid polymorphism in the human genome. Within genomic regions that show evidence of recent hitchhiking, we find fewer neutral but a similar number of deleterious SNPs compared to other genomic regions. The higher ratio of deleterious to neutral SNPs is consistent with simulated hitchhiking events and implies that positive selection eliminates some deleterious alleles and increases the frequency of others. The distribution of disease-associated alleles is also altered in hitchhiking regions. Disease alleles within hitchhiking regions have been associated with auto-immune disorders, metabolic diseases, cancers, and mental disorders. Our results suggest that positive selection has had a significant impact on deleterious polymorphism and may be partly responsible for the high frequency of certain human disease alleles.

## Author Summary

Deleterious mutations reduce fitness within natural populations and must be continually removed by natural selection. However, some deleterious mutations reach unexpectedly high frequencies. There are a number of mechanisms by which this could occur, including changes in genetic or environmental constraints. Here, we investigate the hypothesis that some deleterious mutations have hitchhiked to high frequency due to linkage to sites that have been under positive selection. Using a collated set of regions likely to have been influenced by positive selection, we find that the number of deleterious polymorphisms in hitchhiking and non-hitchhiking regions is similar, but that the ratio of deleterious to neutral polymorphism is higher in hitchhiking compared to non-hitchhiking regions. Both computer simulations and empirical data indicate that while hitchhiking eliminates many deleterious mutations, some are increased in frequency. The distribution of human disease-associated mutations is also altered in hitchhiking compared to non-hitchhiking regions. Together, our results provide evidence that hitchhiking has influenced the frequency of linked deleterious mutations in humans, implying that the evolutionary dynamics of advantageous and deleterious mutations may often depend on one another.

## Introduction

The continuous removal of deleterious mutations is essential to maintaining a species' reproductive output and even its existence. While deleterious mutations incur a considerable fitness cost [1], they are not always effectively removed from a population. Deleterious mutations are more difficult to remove from small populations and their accumulation can lead to further reductions in population size and eventually to extinction, a process called mutational meltdown [2]–[4]. Sexual recombination facilitates the elimination of deleterious mutations [5] and the lack of recombination on the *Y* sex chromosome may have contributed to its degeneration through the accumulation of deleterious mutations [6].

In humans, many deleterious mutations have reached high population frequencies. Each human is estimated to carry on the order of 1,000 deleterious mutations in their genome [7]–[9]. Although most deleterious mutations are rare, a significant fraction is common in the population. For example, 19% of deleterious mutations identified in three human genomes are common enough to be shared among them [9]. However, the cause and consequence of common deleterious mutations have been difficult to determine.

A number of factors may contribute to the large number of common deleterious mutations in humans. Most genome-wide methods used to identify deleterious mutations are based on the alteration of sites that are significantly conserved across species [10], [11]. As such, lineage-specific changes in selective constraint provide one explanation for common alleles that alter highly conserved sites.

Changes in selective constraint can be caused by changes in population size, the environment, or other genetic changes [12]. Because the efficacy of selection is a function of effective population size, a reduction in population size can result in reduced constraints on sites that are conserved in other species [13]. Many common deleterious mutations in humans can be attributed to the small effective population size of humans and recent human population bottlenecks [14], [15]. However, changes in constraint can also be mediated by genetic or environmental changes. For example, the thrifty gene hypothesis posits that the high frequency of diabetes risk alleles is a consequence of their being previously advantageous during periods of food scarcity [16]. Relaxed constraints may also arise due to certain types of genetic changes, such as gene duplication or compensatory mutations. The observation that human disease alleles are often present in mouse supports the notion that the selective constraints on a site are not always static but can change with the genetic or environmental background [17]. However, not all common deleterious mutations may result from species-specific differences in selective constraint.

Positive selection can influence the frequency of deleterious mutations directly, through genetic hitchhiking, or indirectly, through a reduction in effective populations size mediated by an increase in the variance of reproductive success [18]. As a consequence, positive selection can increase the rate at which deleterious mutations accumulate, particularly when the effect of the advantageous mutation outweighs the effects of linked deleterious mutations [19]–[22]. Hitchhiking of deleterious mutations along with advantageous mutations may have contributed to the degeneration of the *Y* sex chromosome [20], [23] and the increased number of deleterious mutations present in domesticated species [24], [25].

In this study, we examined the effect of positive selection on linked deleterious polymorphism in the human genome. We compared the abundance of deleterious and neutral nonsynonymous single nucleotide polymorphisms (SNPs) in regions showing evidence of hitchhiking to other genomic regions. While hitchhiking is expected to remove neutral variation from a population [26], we find that the rate of deleterious SNPs is not reduced, resulting in an enrichment of deleterious relative to neutral SNPs in hitchhiking regions. Our results imply that positively selected mutations may often influence the frequency of linked deleterious mutations.

## Results

### Simulated effect of hitchhiking of deleterious mutations

**Figure 1:**
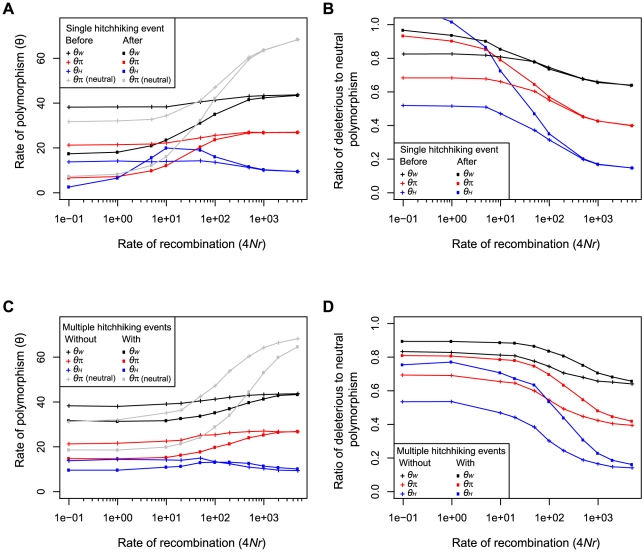
The effect of hitchhiking on neutral and deleterious polymorphism as a function of the rate of recombination. The rate of low, intermediate and high frequency deleterious polymorphism measured by *θ_W_* (black), *θ_π_* (red) and *θ_H_* (blue), respectively, before (crosses) and after (squares) a single hitchhiking event (A) and in the presence (crosses) and absence (squares) of multiple hitchhiking events (C). Average heterozygosity (*θ_π_*) of neutral polymorphism is shown in gray. The ratio of deleterious to neutral polymorphism is shown before (crosses) and after (squares) a single hitchhiking event (B) and in the absence (crosses) and presence (squares) of multiple hitchhiking events (D). All panels show the mean of 500 simulations for which 4*Nu_n_* = 70, 4*Nu_d_* = 70, 4*Ns_d_* = −10 and 4*Ns_a_* = 100, where *N* is the population size, *u* is the mutation rate, *s* is the selection coefficient, and subscripts *n*, *a* and *d* refer to neutral, advantageous and deleterious mutations. In panel A and B, a single hitchhiking event occurs at the center of the chromosome. In panel C and D, 4*Nu_a_* = 0.5 and multiple hitchhiking events occur across the entire chromosome.

To characterize the effect on positive selection on linked deleterious mutations we conducted simulations under a Wright-Fisher model. Subsequent to a single hitchhiking event, the rate of neutral and deleterious polymorphism was reduced as a function of the rate of recombination (Figure 1A). Despite the overall reduction in the number of deleterious polymorphisms, at intermediate rates of recombination, hitchhiking caused an increase in the number of high frequency deleterious polymorphisms, as measured by *θ_H_* (Figure 1A), similar to its effect on neutral polymorphism [27]. Compared to deleterious polymorphism, hitchhiking caused a greater reduction in neutral polymorphism, resulting in an enrichment of deleterious relative to neutral polymorphism. The enrichment was greatest for high compared to intermediate and low frequency polymorphism, as measured by *θ_H_*, *θ_π_*, and *θ_W_*, respectively (Figure 1D). Because the reduction in fitness due to deleterious polymorphism remained constant during hitchhiking, the cost of increasing the frequency of some deleterious alleles to high frequency must be offset by the elimination of other deleterious alleles.

To examine the average effect of multiple hitchhiking events we also simulated populations under a continuous influx of advantageous mutations. Similar to single hitchhiking events, recurrent hitchhiking reduced the rate of neutral and deleterious polymorphism (Figure 1C), and increased the ratio of deleterious to neutral polymorphism (Figure 1B). While the degree to which hitchhiking caused an enrichment of deleterious polymorphism depended on the strength of positive and negative selection and the rate of advantageous and deleterious mutation (Figure S1), our simulations indicate that hitchhiking may often have a measurable impact on the ratio of deleterious to neutral polymorphism segregating in natural populations.

### Classification of deleterious and neutral nonsynonymous SNPs in humans

To examine the impact of positive selection on deleterious polymorphism in humans we classified nonsynonymous SNPs from the 1000 Genomes Project [28] as neutral or deleterious using a likelihood ratio test based on cross-species conservation (Materials and Methods). Although not all classifications may be correct, the likelihood ratio test classifies 72% of human disease mutations as deleterious and only 6.7% of nonsynonymous substitutions between species as deleterious [9]. Out of 48,558 autosomal nonsynonymous SNPs tested, 14,094 (29.0%) were predicted to be deleterious, of which 2,263 (16.1%) have a derived allele frequency of over 10%. Using a cutoff of 10%, the fraction of SNPs called deleterious is 17.8% for common alleles compared to 33.0% for rare alleles, consistent with the expected effects of negative selection.

### Enrichment of deleterious SNPs in regions showing evidence of hitchhiking

**Figure 2:**
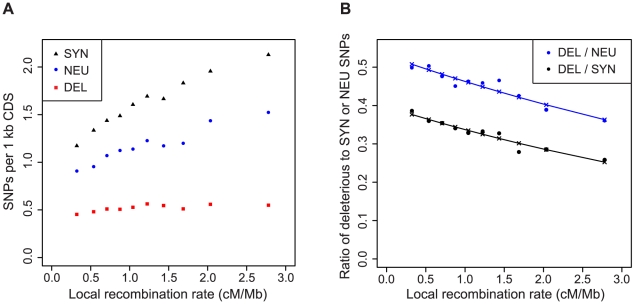
Regions of low recombination are enriched for deleterious SNPs. The number of synonymous (SYN) and neutral nonsynonymous (NEU) and deleterious (DEL) SNPs per kb of coding sequence (A) and the ratio of deleterious to synonymous or neutral nonsynonymous SNPs (B) as a function of the local recombination rate. The rate of neutral and deleterious SNPs was normalized by the number of sites that were testable by the likelihood ratio test. Lines show the results of logistic regression.

Hitchhiking is expected to have a stronger effect on linked variation in regions of low recombination [26]. While the spread of a positively selected allele through a population causes a reduction in the amount of linked neutral variation, it may interfere with the elimination of linked deleterious mutations. Consistent with this hypothesis, the rate of synonymous and neutral nonsynonymous SNPs decreases in regions of low recombination, whereas the rate of deleterious SNPs remains nearly constant (Figure 2A). As a consequence, the ratio of deleterious to neutral and deleterious to synonymous SNPs is significantly correlated with the rate of recombination (P = 3.1×10^−15^ and P<2.0×10^−16^, respectively, Figure 2B). The association remains significant when accounting for the frequency of conserved codons and biased gene conversion (P = 2.1×10^−7^ and P = 9.3×10^−6^, respectively, Figure S2), which are also correlated with the rate of recombination. However, this correlation is also expected due to background selection, which reduces the efficacy of selection against deleterious mutations [29], [30].

In contrast to background selection, which exerts more uniform effects across the genome [31], hitchhiking can generate strong local effects. Furthermore, hitchhiking can have large effects in regions of both low and high recombination whereas background selection is expected to have much smaller effects in regions of high recombination [32].

**Figure 3:**
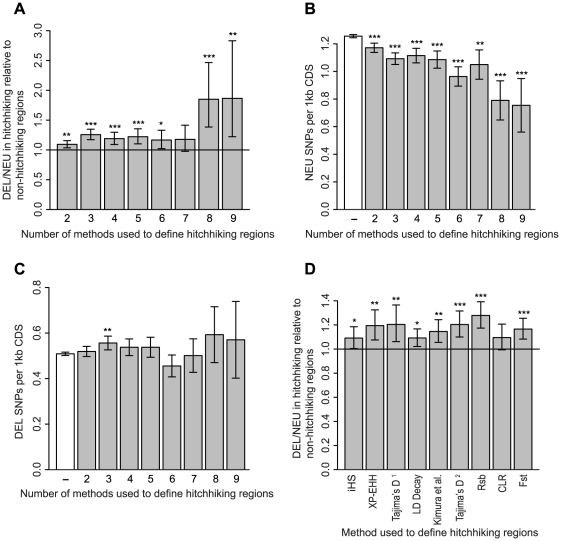
Rates of deleterious and neutral SNPs in hitchhiking and non-hitchhiking regions. The ratio of deleterious (DEL) to neutral (NEU) SNPs is higher in hitchhiking relative to non-hitchhiking regions (A). The rate of neutral SNPs is reduced (B) and the rate of deleterious SNPs remains relatively constant (C) in hitchhiking compared to non-hitchhiking regions. The x-axes in panels A–C denotes the minimum number of methods used to define hitchhiking regions. Non-hitchhiking regions are labeled by a dash (–). The ratio of deleterious to neutral SNPs is higher in hitchhiking to non-hitchhiking regions for the majority of tests of selection (D). Tajima's D was used by two studies: [76] ^1^ and [77] ^2^. Bars show 90% confidence intervals, one, two and three stars indicate P<0.05, P<0.01, and P<0.001 based on a one-sided Fisher's Exact Test.

To determine whether deleterious SNPs have been influenced by recent episodes of positive selection, we examined genomic regions showing evidence of hitchhiking based on multiple tests of selection [33]. In hitchhiking regions defined by two or more tests of selection, we found a significantly higher ratio of deleterious to neutral SNPs compared to other genomic regions (Figure 3 and Table S1). The elevated ratio of deleterious to neutral SNPs within hitchhiking regions cannot be explained by a reduced rate of recombination or a higher density of conserved sites; the difference between hitchhiking and non-hitchhiking regions remained significant using a logistic regression model with these factors as covariates (P = 6.3×10^−5^, Figure S3). The increase in the ratio of deleterious to neutral SNPs in hitchhiking relative to non-hitchhiking regions is 1.09-fold for regions identified by two or more tests of selection and increases to 1.87-fold for regions identified by all nine tests of selection. The increase in the ratio of deleterious to neutral SNPs in hitchhiking regions is due to a decrease in the number of neutral SNPs rather than an increase in the number of deleterious SNPs (Figure 3B and 3C). With the exception of the composite likelihood ratio test (CLR) [34], all of the methods used to detect hitchhiking identify regions with a higher ratio of deleterious to neutral SNPs (Figure 3D). Thus, the increase in the relative abundance of deleterious SNPs in hitchhiking regions does not appear to be associated with any specific test of selection.

**Figure 4:**
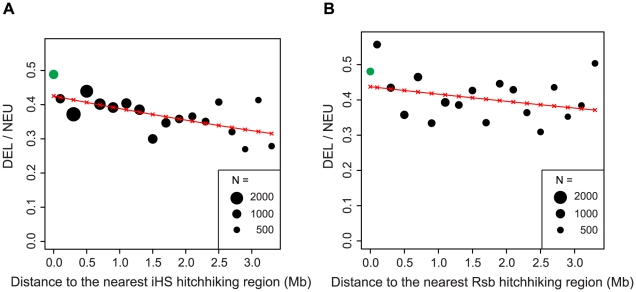
The ratio of deleterious to neutral nonsynonymous SNPs declines as a function of distance to the nearest hitchhiking region. Hitchhiking regions were defined using the European population by iHS (A) or Rsb (B). Sample size is indicated by circle size. Green circles represent iHS and Rsb hitchhiking regions.

The effects of hitchhiking are expected to decline as a function of recombinational distance from the site under selection [26]. To examine the decay in the number of deleterious SNPs associated with hitchhiking, we used iHS [35] and Rsb [36] defined hitchhiking regions. iHS is better at detecting incomplete hitchhiking events [35], where the advantageous mutations is still segregating in the population, whereas Rsb is better at detecting complete or nearly-complete episodes of selection [36]. The frequency of deleterious SNPs decreases as a function of distance from iHS defined hitchhiking region (P = 2×10^−7^, Figure 4A). Compared to iHS regions, the frequency of deleterious SNPs shows a more modest decline with distance from the Rsb defined hitchhiking regions (P = 0.018, Figure 4B). This difference could result from Rsb detecting older hitchhiking events providing additional time for negative selection to eliminate linked deleterious mutations or due to a weaker influence of hitchhiking outside of Rsb defined regions, which are twice as large as iHS defined regions (Table S1).

### Hitchhiking regions show a similar enrichment of rare, intermediate, and common deleterious SNPs

As the rate of recombination decreases, hitchhiking causes a larger increase in the ratio of deleterious to neutral SNPs for common compared to low frequency SNPs (Figure 1). To determine whether hitchhiking regions show a similar pattern, we compared the ratio of deleterious to neutral SNPs as a function of allele frequency. Similar to the simulation results, the ratio of deleterious to neutral SNPs declines with increasing allele frequency. However, the ratio of deleterious to neutral SNPs in hitchhiking regions is not significantly different among three frequency classes (Table 1). We observed the same pattern using HapMap SNPs (data not shown) indicating that low coverage sequencing errors in the 1000 Genomes Project is unlikely to explain this result. Although the absence of a difference in the ratio of deleterious to neutral SNPs across allele frequencies is somewhat surprising, it is consistent with simulations that have a high rate of recombination or strong negative selection (Figure 1 and Figure S1).

**Table 1:** The ratio of deleterious to neutral SNPs at different allele frequencies.

	Deleterious/Neutral		
Allele frequency	Hitchhiking	Non-hitchhiking	Fold increase in hitchhiking regions(95% CI)
Rare (0–0.008)	650/1013 (0.64)	5469/9109 (0.60)	1.07 (0.96–1.19)
Intermediate (0.008–0.059)	470/1050 (0.45)	4241/10376 (0.41)	1.10 (0.97–1.23)
Common (0.059–1.0)	329/1206 (0.27)	2935/11710 (0.25)	1.09 (0.95–1.24)

Hitchhiking regions are defined by two or more tests of selection.

### Deleterious SNPs in regions showing population-specific patterns of hitchhiking

Many of the methods used to detect hitchhiking were independently applied to populations of different ancestry. Although some hitchhiking events may be specific to European, African, or Asian populations, e.g. [35], the power to detect hitchhiking is expected to differ among populations even when an adaptive mutation is fixed in all populations [37], [38]. We examined the enrichment of deleterious SNPs in iHS defined hitchhiking regions in the European, African, and Asian samples. Surprisingly, we found no enrichment of deleterious SNPs in African and Asian defined hitchhiking regions (Table S2). Despite these population-specific differences revealed by iHS, the ratio of deleterious to neutral SNPs is elevated in hitchhiking regions defined by multiple methods in the African, European and Asian samples (Table S3).

### Deleterious SNPs within and around genes under positive selection

For most hitchhiking regions the target of selection is not known. We identified ten hitchhiking regions from the literature for which there is evidence for the target of selection. The putative targets are *LCT* [39], [40], *SLC45A2* [41], *TYRP1* [42], *HERC2* [42], *KITLG* [42], *SLC24A5* [43], *TYR* [44], *EDAR* [41], *PCDH15* [42] and *LEPR* [42]. Within these genes the ratio of deleterious to neutral SNPs (1.83) is higher than in non-hitchhiking regions (0.41) (Fisher's Exact Test P = 0.0023, Table 2). The deleterious SNPs include 5/6 nonsynonymous SNPs that are putative targets of selection. Within the 1 Mbp regions flanking these genes, there is also a higher ratio of deleterious to neutral SNPs (0.69) relative to that in non-hitchhiking regions (0.41) (Fisher's Exact Test P = 0.034).

**Table 2:** Deleterious SNPs within and around genes under positive selection.

	Within target gene		Target gene +/− 1 Mbp of flanking region		
Putative target of selection	Deleterious^1^	Neutral	Deleterious^1^	Neutral	Genes with deleterious SNPs^2^
*LCT*, noncoding (lactose tolerance)	1	2	4	6	*R3HDM1 (2), LCT, MCM6*
*SLC45A2*, L374F (pigmentation)	1 (1)	0	3 (1)	8	*ADAMTS12, SLC45A2, C1QTNF3*
*TYRP1*, noncoding (pigmentation)	0	0	1	3	*MPDZ*
*OCA2-HERC2*, noncoding (pigmentation)	3	2	3	2	*OCA2 (3)*
*KITLG*, noncoding (pigmentation)	0	0	1	5	*CEP290*
*SLC24A5*, A111T (pigmentation)	1 (1)	0	2 (1)	3	*SLC24A5, SLC12A1*
*TYR*, S192Y (pigmentation)	2 (1)	0	6 (1)	1	*GRM5, TYR (2), FOLHB1 (3)*
*EDAR*, V370A (thicker hair)	1 (1)	1	2 (1)	5	*GCC2, EDAR*
*PCDH15*, D435A (unknown)	0	0	0	0	
*LEPR*, K109R (metabolism)	2 (1)	1	2 (1)	2	*LEPR(2)*
Total	11 (5)	6	24 (5)	35	

The number of deleterious and neutral nonsynonymous SNPs were tabulated using the European sample (CEU), except for *EDAR*, *PCDH15* and *LEPR*, which were tabulated using the Asian sample (CHB+JPT). A111T in *SLC24A5*, one deleterious SNP in *OCA2*, and three neutral SNPs in flanking regions of *LCT*, *SLC45A2*, and *SLC24A5* are fixed or nearly fixed in CEU.

1 Putatively functional nonsynonymous SNPs under selection are in parentheses.

2 The number of deleterious SNPs within flanking genes is in parentheses if greater than one.

Positive selection at *SLC45A2* and *TYR* is particularly interesting since linked deleterious SNPs have been associated with human disease. The putative target of selection on *TYR* is a nonsynonymous SNP (S192Y) that has an allele frequency of 42% in the European sample (CEU) and is associated with the absence of freckles in Europeans [44]. Another nonsynonymous SNP in *TYR* (R402Q), 106 kb away, is classified as deleterious, has a frequency of 21% in CEU and is associated with mild ocular albinism and risk for cutaneous melanoma and basal cell carcinoma [45], [46]. The putative target of selection on *SLC24A5* is a nonsynonymous SNP (A111T) that is associated with skin pigmentation and is nearly fixed in European populations but is at low frequency in African and Asian populations [43]. Positive selection on this allele may have influenced the frequency of deleterious SNPs in *FBN1*, 265 kb downstream of *SLC24A5*. *FBN1* has five deleterious SNPs in HapMap CEU, all of which are present at low frequency in CEU, 0.5–1.4%, but are absent from both the African or Asian HapMap samples. Three of these deleterious SNPs cause Marfan syndrome [47], [48] and one has been found in patients with Marfan syndrome or related phenotypes [49].

### Disease-associated alleles within hitchhiking regions

Hitchhiking may have also influenced SNPs that are associated with human disease. This might occur by increasing the frequency of rare, disease-causing mutations or by increasing the frequency of more common, disease-risk alleles. To investigate this possibility we compared the abundance of disease-associated alleles in hitchhiking and non-hitchhiking regions.

**Figure 5:**
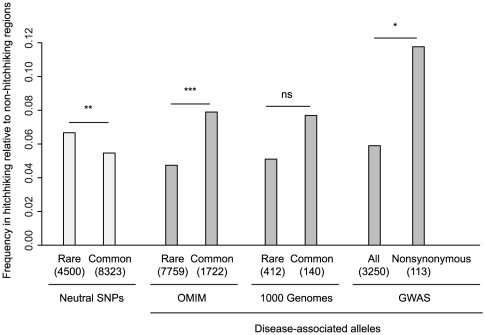
Enrichment of disease-associated alleles in hitchhiking relative to non-hitchhiking regions. Each category shows the number within hitchhiking to non-hitchhiking regions, where hitchhiking regions were defined by the overlap of three or more tests of selection. Neutral SNPs are from HapMap Phase II. Disease alleles in 1000 Genomes columns are based on the Human Gene Mutation Database. The sample size of each category is shown in parentheses. Bars show one-sided Fisher's Exact test comparisons, not significant (ns), P<0.05 (*), P<0.01 (**), and P<0.001 (***).

Within known disease genes in OMIM, there are 9,481 mutations that have been associated with human disease, of which 1,722 were common enough to be typed in the HapMap project and can be considered SNPs. The ratio of all OMIM variants in hitchhiking relative to non-hitchhiking regions (0.053) is lower than that of the number of OMIM morbid genes (0.071), consistent with the elimination of variation within hitchhiking regions (Table S4). However, the ratio of common OMIM variants in hitchhiking to non-hitchhiking regions, 0.079, is significantly higher than that of rare variants, 0.047 (Fisher's Exact Test, P<10^−5^, Figure 5). This difference is opposite to that found for neutral HapMap SNPs, which are skewed towards rare alleles in hitchhiking relative to non-hitchhiking regions. Furthermore, the minor allele frequencies of OMIM SNPs is slightly higher in hitchhiking compared to non-hitchhiking regions (Wilcoxon Rank Sum Test, P = 0.03). Similar to OMIM SNPs, the ratio of disease-associated SNPs in hitchhiking relative to non-hitchhiking is higher for common compared to rare alleles identified in the 1000 Genomes Project, although the difference is not significant (Figure 5, Fisher's Exact Test, P = 0.20). For the 1000 Genomes Project data, the mean frequency of common disease alleles in hitchhiking regions (0.25) is higher than that in non-hitchhiking regions (0.20), although the difference is not significant (Wilcoxon Rank Sum Test, P = 0.80). Thus, hitchhiking regions appear to be characterized by an increase in the number common disease-associated SNPs rather than by an increase in the number of rare, disease-associated variants.

To examine the abundance of common, risk-associated alleles within hitchhiking regions, we used alleles that have been associated with human disease from genome-wide association studies (GWAS) [50] and from a literature survey (see Materials and Methods). Consistent with a previous study [50], the ratio of risk-alleles identified by GWAS in hitchhiking to non-hitchhiking regions, 0.059, is not greater than that expected based on the number of genes, 0.068 (Table S4). However, nonsynonymous risk alleles, which are likely enriched for functional variants, have a higher hitchhiking to non-hitchhiking ratio than that of other risk-alleles (Figure 5, Fisher's Exact Test, P = 0.02). Although risk alleles in hitchhiking regions do not have significantly higher allele frequencies than those in non-hitchhiking regions (Wilcoxon Rank Sum Test, P = 0.63), the proportion of risk alleles with odds ratios over 2.0 in hitchhiking regions (18.9%) is significantly higher than that in non-hitchhiking regions (11.5%) (Fisher's Exact Test, P = 0.03). For disease-associated nonsynonymous SNPs identified in a literature survey, the ratio of SNPs in hitchhiking to non-hitchhiking regions is lower than that of neutral SNPs (Table S4).

### Disease-phenotype classification

To identify which types of diseases hitchhiking may have influenced, we examined disease-associated SNPs and genes with deleterious SNPs within hitchhiking regions. Classification of the 126 OMIM SNPs within hitchhiking regions by phenotype (Table S5) revealed a number of SNPs involved auto-immune disorders (21 SNPs), energy metabolism (16 SNPs), and a variety of mental, neurological, and neurodevelopmental disorders (25 SNPs). Classification of the 461 genes (Table S6) within hitchhiking regions that contain deleterious SNPs by their disease association revealed a number that have been associated with cardiovascular (N = 21), immune (N = 19), metabolic (N = 18), neurological (N = 12) and psychiatric disease (N = 10), and cancer (N = 17), according to the Genetic Association Database classification [51]. Classification of the 12 nonsynonymous SNPs identified by GWAS and the three nonsynonymous SNPs identified from the literature revealed five associated with auto-immune disease, three associated with metabolic disease, and two associated with cancer. However, none of these disease classifications are significantly different from those outside of hitchhiking regions.

### Genome clustering of deleterious SNPs

Most deleterious SNPs lie outside of currently defined hitchhiking regions. However, this does not exclude the possibility that they were influenced by positive selection. The overlap among methods used to detect hitchhiking is low [33], and some hitchhiking events may not be detected by any of the methods. For example, a beneficial mutation may initially spread slowly through a population while it becomes disentangled from linked deleterious mutations. In this scenario, patterns of hitchhiking may be weak or absent, similar to those that occur when positive selection acts on standing genetic variation [52]. To characterize genomic regions enriched for deleterious SNPs, we split the genome into 1 Mbp windows and selected the top 2% of windows with the highest rate of deleterious SNPs per kb of coding sequence.

Regions enriched for deleterious SNPs have a high ratio of deleterious to neutral nonsynonymous SNPs, 0.66, much higher than the genome average, 0.41. Together, these 43 regions contain 7.4% of deleterious SNPs (Table S7). 17 of these regions show evidence of hitchhiking, ten with evidence from three or more tests of selection. In addition, one region may have been influenced by positive selection on *DARC* [53], even though it does not overlap with hitchhiking regions defined by multiple tests of selection [33]. Ten of the regions contain deleterious SNPs in multiple duplicated olfactory receptor or keratin genes. Of the remaining 21 regions, 16 have deleterious SNPs in more than two genes. While loss of constraint may explain the accumulation of deleterious SNPs in some genes, particularly those that are duplicated, it is less likely to explain deleterious SNPs in multiple linked genes with disparate functions.

## Discussion

Deleterious mutations have a significant impact on a species' ability to survive, reproduce and adapt to new environments [2]–[4]. In humans, there is an abundance of common nonsynonymous SNPs that disrupt sites highly conserved across species and likely to be deleterious [9]. By examining the genome distribution of nonsynonymous SNPs classified as either neutral or deleterious, we found a greater reduction in neutral compared to deleterious polymorphism within genomic regions likely to have been influenced by hitchhiking. This observation combined with hitchhiking simulations suggests that while many deleterious SNPs are eliminated due to hitchhiking, a substantial number of rare deleterious mutations must also increase to frequencies common enough to be considered polymorphic. Our results imply that positive selection is not responsible for the abundance of common deleterious SNPs across the human genome but is relevant to understanding the distribution and dynamics of deleterious mutations as well as certain disease alleles.

Despite evidence for a hitchhiking effect, most common deleterious SNPs are unlikely to have been influenced by positive selection and are better explained by a change in selective constraint, mediated by a population bottleneck [15] or environment change [54]. Only 11.5% of deleterious SNPs occur in regions showing evidence of hitchhiking (Table S1). However, this does not exclude the possibility that positive selection has influenced the frequency of some deleterious SNPs outside of hitchhiking regions. Hitchhiking regions were defined by the overlap of two or more methods of detecting selection and are unlikely to include all regions influenced by hitchhiking [33]. In support of this possibility, we identified a number of genomic regions that contain an exceptionally high ratio of deleterious to neutral SNPs. Although some of these regions include multiple duplicated genes, which could explain the large number of SNPs predicted to be deleterious, one of the regions includes a gene thought to have been under selection, *DARC* [53], and many of the regions contain deleterious SNPs in genes with disparate functions.

Within hitchhiking regions, we found an elevated ratio of deleterious to neutral SNPs caused by a reduction in the number of neutral SNPs. The elevated ratio of deleterious to neutral SNPs is consistent with simulations of both single and recurrent hitchhiking events across a range of parameters (Figure 1 and Figure S1) and can be explained by the difference in the frequency distribution of deleterious and neutral SNPs prior to hitchhiking. During a hitchhiking event neutral and deleterious alleles increase or decrease in frequency depending on their original configuration with the advantageous mutation. However, rare alleles are more likely to be deleterious and common alleles are more likely to be neutral. Thus, positive selection removes many common alleles, which tend to be neutral, and increases the frequency of many rare alleles, which tend to be deleterious, resulting in an increase in the ratio of deleterious to neutral SNPs. However, the simulated hitchhiking events showed two patterns that were not observed in the human data. First, hitchhiking caused a reduction in the number of deleterious SNPs. Second, hitchhiking caused a much larger increase in the ratio of deleterious to neutral SNPs at high frequencies relative to that at low frequencies. The significance of these differences is hard to evaluate since many factors known to influence hitchhiking were not examined, e.g. dominance, population structure, changes in population size and selection on new mutations versus standing genetic variation. Furthermore, hitchhiking simulations with high rates of recombination or strong selection against deleterious mutations tended to show patterns that are more consistent with those observed in humans (Figure 1 and Figure S1). Although some theoretical results have recently been obtained [22], further work will be needed to understand the effects of hitchhiking on deleterious mutations in humans.

A number of factors besides hitchhiking may contribute to the increased ratio of deleterious to neutral SNPs. Background selection is expected to increase the ratio of deleterious to neutral SNPs, particularly within regions of low recombination (Figure 1). While the rate of recombination can explain some of the difference between hitchhiking and non-hitchhiking regions, the ratio of deleterious to neutral SNPs is significantly higher in hitchhiking regions even after controlling for differences in recombination rate between hitchhiking and non-hitchhiking regions. Given the slightly lower rates of recombination in hitchhiking regions, the logistic regression model predicts hitchhiking regions should have a ratio of deleterious to neutral SNPs of 0.46, which is only slightly higher than that in non-hitchhiking regions, 0.44, and less than that observed, 0.53. It is conceivable that background selection may exert much weaker effects over shorter intervals that are not related to regional rates of recombination. However, weak background selection would have to exert a stronger influence within hitchhiking compared to non-hitchhiking regions, making it difficult to attribute the increased ratio of deleterious to neutral SNPs within these regions to background selection alone.

Another factor that complicates the analysis of differences between hitchhiking and non-hitchhiking regions is how hitchhiking regions were defined. Hitchhiking regions were defined by genome scans for patterns of variation expected to occur as a result of positive selection. However, some regions identified in genome scans for selection are likely neutral outliers that by chance show patterns of variation similar to those created by hitchhiking. This was one of our main motivations for using hitchhiking regions defined by two or more genome scans for selection. Although a contribution from neutral outliers cannot be excluded, the observation that the ratio of deleterious to neutral SNPs is 1.87-fold higher in regions identified by all nine genome scans and 1.68-fold higher in regions containing genes known to have been under positive selection suggests that hitchhiking makes a significant contribution to the elevated ratio of deleterious to neutral SNPs.

Similar to deleterious SNPs, common, disease-associated SNPs are enriched in hitchhiking compared to non-hitchhiking regions. In contrast, the number of rare, disease-associated mutations in hitchhiking relative to non-hitchhiking regions is lower than that of OMIM morbid genes. This difference can be explained by hitchhiking. Since most rare disease mutations occur on different chromosomes, hitchhiking will increase the frequency of one or a small number of disease mutations but decrease or eliminate the majority of rare disease mutations. However, the difference between rare and common disease-associated alleles is complicated by the heterogeneous evidence used to define disease-associated mutations in OMIM and the fact that common mutations are more likely to be associated with disease than rare mutations. The effect of hitchhiking on GWAS SNPs is more complex since most GWAS SNPs may be neutral. The ratio of GWAS SNPs in hitchhiking to non-hitchhiking regions is lower than that of all genes or neutral SNPs (Table S4). The lower frequency of GWAS SNPs in hitchhiking to non-hitchhiking regions is consistent with a previous study [50] and may be caused by the removal of common SNPs and reduced power of linkage disequilibrium-based tests of association. Consistent with this possibility, the hitchhiking to non-hitchhiking ratio of GWAS SNPs that are nonsynonymous, and thus more likely to be causative, is higher than that of all GWAS SNPs.

Our results also bear on the incidence of certain human diseases [55], [56] and disease alleles [57], which in some cases are higher than what one might expect based on disease severity. While genetic drift and population bottlenecks are likely to contribute to common disease alleles, balancing selection has also been invoked in some instances. For example, the high frequency of the delta F508 mutation in *CFTR* has been hypothesized to be the result of a heterozygote advantage due to cholera resistance [58], [59]. Mutations in *G6PD* and *Beta-globin* have been hypothesized to provide a heterozygote advantage due to malaria resistance [57]. Another explanation for why some disease alleles are so common is the ancestral-susceptibility hypothesis, under which derived alleles associated with human disease were advantageous to ancestral lifestyles and environmental conditions [54]. Similarly, under the less is more model, loss of function mutations that were previously disadvantageous can become advantageous [60]. In support of this model, we found five out of six nonsynonymous SNPs that are putative targets of positive selection are highly conserved across species and so classified as deleterious.

However, our results also provide evidence for an alternative explanation for the frequency of common disease-associated alleles: the frequency of certain disease alleles is increased due to hitchhiking with linked advantageous mutations. A number of previous observations support this explanation. The MHC locus has been associated with over 40 human genetic diseases [61], and multiple lines of evidence suggest long-term balancing selection [62]. A mutation in *HFE* that causes hemochromatosis is 150 kb away from a hitchhiking region and may have increased in frequency due to hitchhiking [63]–[65]. Hitchhiking has also been implicated in the increased frequency of a common risk haplotype for diabetes, hypertension and celiac disease [66] and another risk haplotype for Crohn's disease [67]. Intriguingly, the delta F508 mutation in *CFTR* is one of the most common disease-causing alleles in Caucasians, with an estimated allele frequency of 1.4% [68], and *CFTR* occurs within a hitchhiking region. Four of the HapMap nonsynonymous SNPs within *CFTR* are classified as deleterious, one of which has been associated with infertility [69]. One of the regions with the strongest evidence for hitchhiking (7 tests) also has one of the highest ratios of deleterious to neutral SNPs (16/22, Table S7). Within this region, 8/16 deleterious SNPs occur in *BLK*, *NEIL2*, and *CTSB*, and there are three disease alleles in the Human Gene Mutation Database [70], with frequencies of 0.8%, 5.3% and 44% based on the 1000 Genomes Project. The frequency of these deleterious/disease alleles may have been influenced by positive selection in this region.

The interaction between positive and negative selection makes it difficult to isolate and understand the effects of each individually. In the presence of deleterious mutations, the effect of hitchhiking on linked neutral variation may be reduced compared to that which would occur in the absence of deleterious mutations, similar to patterns created by soft sweeps [52]. Conversely, hitchhiking increases the frequency of some deleterious mutations and decreases the frequency of others such that the distribution of deleterious mutations is significant different from that expected in the absence of hitchhiking. Furthermore, the recent expansion in human population size combined with population subdivision may amplify or reduce the influence of hitchhiking on deleterious SNPs. This will make it valuable to examine the extent to which deleterious alleles are enriched in hitchhiking regions in other species, particularly domesticated species where the strength of selection was likely strong and for which targets of selection are in some cases known.

## Materials and Methods

### Computer simulations

The effects of hitchhiking on deleterious and neutral polymorphism were simulated using a Wright-Fisher model [71]. Simulated populations had a size, *N*, of 1000 diploid individuals. Mutations were distributed into the population assuming an infinite sites model with a Poisson rate of 2*Nu*, where *u* is the mutation rate per chromosome. A Poisson number of recombination events was generated in the population with a rate of *Nr*, where *r* is the rate of recombination per individual. Chromosomes in the next generation were sampled based on the fitness of the individual from which they were derived. Fitness was calculated by the multiplicative effects of each non-neutral allele, 1+*hs* for heterozygous sites and 1+*s* for homozygous sites, where *s* is the selection coefficient and *h* is the degree of dominance. The dominance coefficient was 0.5 for all simulations. For each set of parameters, simulations were run for 20*N* generations before sampling. For a single hitchhiking event, an advantageous mutation was generated in the center of the chromosome and sampled at the end of hitchhiking conditional on its fixation. For multiple hitchhiking events, advantageous mutations were generated at a constant rate uniformly across the chromosome and samples were taken in intervals of *N* generations. *θ_W_*, *θ_π_* and *θ_H_* were estimated using a sample size of 100 chromosomes as described in [27].

### Classification of neutral and deleterious SNPs

Low-coverage SNP calls for CEU, CHB+JPT, and YRI samples were downloaded from the 1000 Genomes Project (release 2010_07) [28], and all tri-allelic sites were filtered out. Coding SNPs were identified based on their genomic coordinates in the NCBI reference genome (build 36) and Ensembl known genes (release #49). After eliminating SNPs on the sex chromosomes, SNPs in known pseudogenes or gene fragments, and sites monomorphic across CEU, CHB+JPT and YRI samples, there were 47,730 synonymous and 48,558 nonsynonymous SNPs within coding regions with multi-species alignments used by the likelihood ratio test (see below).

Nonsynonymous SNPs were classified as neutral or deleterious using a previously implemented likelihood ratio test (LRT) for conservation across multiple species [9]. The LRT is based on 18,993 multiple sequence alignments from 32 vertebrate species. Positions with less than 10 aligned eutherian mammals were excluded from the analysis due to low power of the LRT. At each codon in the alignment, the LRT calculates the likelihood of the data under a neutral model, where the nonsynonymous substitution rate (*dN*) equals the synonymous substitution rate (*dS*), relative to a conserved model, where *dN* can deviate from *dS*. For these calculations, *dS* is set to an average rate of 12.2 substitutions per site across the entire tree based on an estimate from gap-free concatenated alignments of 1,227 genes (54 kb) with data from all species. Nonsynonymous SNPs were predicted to be deleterious if: 1) the codon is significantly conserved by the LRT (P<0.001), 2) *dN* is less than *dS*, and 3) the derived amino acid is not present at orthologous positions in other eutherian mammals.

### Correlation of SNP density with recombination rate

The density of SNPs was measured as a function of local recombination rate using CEU, CHB+JPT, and YRI SNPs from the 1000 Genomes Project. Following previous work [72], recombination rates were estimated from non-overlapping 400 kb windows by dividing the genetic map distance of the two most distant SNPs by their physical distance. The genetic map, estimated by LDhat [73], was obtained from the 1000 Genomes Project. Windows that were less than 10 Mb away from the end of centromeres and telomeres, windows without a pair of SNPs greater than 360 kb apart, and windows with no aligned coding sequence were excluded. The remaining 3,666 windows were assigned into ten equal-sized bins by their recombination rates, and the number of synonymous, nonsynonymous deleterious and nonsynonymous neutral SNPs was counted per kb of aligned coding sequence in each bin. To account for the the proportion of codons that are conserved, which is correlated with both the rate of recombination and the number of G or C nucleotides within codon (Figure S2A), codons in each recombination bin were subdivided into four classes by the number of GC nucleotides within the human codon (*j* = 0, 1, …, 3). In cases of polymorphic codons, GC content of the ancestral codon were counted. A total of 6,248,078 codons were classified as significantly conserved or not by the LRT at a P-value cutoff of 0.001. The relationship between recombination and the ratio of deleterious to neutral SNPs was assessed using the logistic regression model:
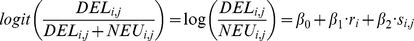
where *DEL_i, j_*, and *NEU_i, j_*, are the number of deleterious and neutral nonsynonymous SNPs, respectively, *r_i_* is the average recombination rate of windows in bin *i*, and *s_i, j_* adjusts for differences in the number of potentially deleterious sites. *s_i, j_* was estimated by:
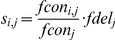
where *fcon_i, j_* is the fraction of conserved codons out of all aligned codons with *j* GC nucleotides in bin *i*, *fcon_j_* is the mean of *fcon_i, j_* over all *i* = 1, …, 10, and *fdel_j_* is the fraction of deleterious out of all tested nonsynonymous SNPs with the same *j* GC nucleotides.

To account for biased gene conversion, which has been previously proposed to explain a higher rate of GC-biased disease alleles in regions of higher recombination [74], we re-examined the relationship between the ratio of deleterious to neutral SNPs and recombination after excluding 13,995 AT-to-GC mutating SNPs potentially affected by biased gene conversion. SNPs within codons with zero GC nucleotides were also eliminated due to their relatively small number (N = 335). Using the logistic regression model that accounts for the variation in the number of potentially deleterious sites, the regression coefficient *β*_1_ of recombination rate remained similar (−0.097 to −0.101) and highly significant (P = 9.3×10^−6^).

### SNPs in hitchhiking and non-hitchhiking regions

Hitchhiking regions were defined by genomic intervals that were identified by two or more out of nine tests for hitchhiking, using intervals rounded to the nearest multiple of 10 kbp [33]. To compare different methods, we examined regions that were identified by one method and overlapped with any other method. Non-hitchhiking regions were defined as autosomal regions excluding hitchhiking regions as defined above. The density of deleterious and neutral nonsynonymous SNPs was measured relative to the accessible portion of aligned coding regions used for the likelihood ratio test. The accessible genome, which satisfies minimum read depth required for SNP calling, was obtained from the 1000 Genomes Project for CEU, CHB+JPT, and YRI [28], and their union was used for the combined analysis of all samples. The difference between the SNP density within hitchhiking and non-hitchhiking regions was tested by a two-proportion z-test.

To test whether a higher ratio of deleterious to neutral SNPs in hitchhiking relative to non-hitchhiking regions is caused by a higher recombination rate or a larger number of potentially deleterious sites in hitchhiking regions, the 400-kb genomic windows which were already binned by the rate of recombination and the number of GC nucleotides in a codon were further classified into hitchhiking and non-hitchhiking groups. After removing windows near centromeres and telomeres, there were 388 windows identified by three or more tests of hitchhiking that were assigned to the hitchhiking group (*h* = 1), and 2,917 windows without any hitchhiking regions that were assigned to the non-hitchhiking group (*h* = 0). The data were fit to the following logistic regression model:
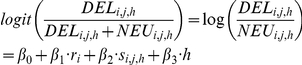
where *r_i_* is the rate of recombination, *s_i,j,h_* adjusts for the density of conserved codons and *h* is an indicator variable for hitchhiking windows.

To study the decay of the ratio of deleterious to neutral SNPs as a function of distance from hitchhiking regions, we used regions identified in CEU by iHS [35] and Rsb [36]. For iHS, the top 5% of scanned genomic windows (a total of 127.6 Mb) were used as hitchhiking regions, as described below. For Rsb, we used regions identified in CEU in comparison to both YRI and CHB+JPT (a total of 119.7 Mb). Deleterious and neutral nonsynonymous SNPs outside iHS and Rsb regions were assigned into bins of non-overlapping 200-kb windows by their distance from the nearest hitchhiking region. The ratio of deleterious to neutral SNPs was modeled as a function of the distance (*d_k_*) of each window in bin *k* to the nearest hitchhiking region using logistic regression:
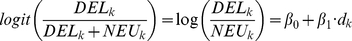



Population specific patterns of hitchhiking were examined using regions identified by multiple tests of selection and by iHS alone. Regions identified by multiple tests of selection were not differentiated by which population showed evidence of selection and so represent a composite view of hitchhiking [33]. iHS regions were identified in CEU, CHB+JPT, and YRI, using empirical cutoffs of 0.25%, 1%, and 5%. To identify iHS hitchhiking regions using HapMap Phase II data, iHS scores of individual SNPs (HapMap Phase II) were downloaded (http://hg-wen.uchicago.edu/selection/), and for each 100-kb non-overlapping genomic window the signal of selection was evaluated by the fraction of SNPs with iHS scores above +2 or below −2, as in Voight et al. [35]. Windows were grouped into bins by the number of SNPs within the window using increments of 25 SNPs. Empirical cutoffs were applied separately to each bin. Windows with less than 10 SNPs and bins with less than 100 windows (less than 400 for the 0.25% cutoff) were excluded.

### Disease-associated alleles in hitchhiking and non-hitchhiking regions

Disease-associated alleles were obtained from OMIM (http://www.ncbi.nlm.nih.gov/omim), a catalog of published GWAS studies (http://www.genome.gov/26525384) and Google Scholar searches of the literature. For OMIM, dbSNP IDs (release #132) with OMIM links were downloaded (ftp://ftp.ncbi.nih.gov/snp/database/organism_data/human_9606/OmimVarLocusIdSNP.bcp.gz). Excluding InDels, unmapped variants, and variants on sex chromosomes, 10,775 OMIM variants were re-mapped to the reference genome using UCSC's LiftOver program. All OMIM variants included in HapMap Phase II (release #24) were considered common enough to be SNPs with the exception of those with minor allele frequency of zero. Average minor allele frequency across CEU, CHB+JPT, and YRI was compared between hitchhiking and non-hitchhiking regions. For allele frequency, HapMap Phase II+III (release #26) data were used [75]. For disease SNPs identified in the 1000 Human Genomes project [28], common and rare variants were distinguished by their mean allele frequencies across CEU, CHB+JPT, and YRI using a 5% allele frequency cutoff. SNPs without allele frequencies were set to an allele frequency of zero.

Disease-risk alleles were obtained from a catalog of published Genome-Wide Association Studies (GWAS) [50]. Excluding 115 regions without associated SNPs and 10 regions with multi-SNP haplotype associations, we obtained 3,383 non-redundant autosomal risk alleles with the strongest trait association at each locus from a total of 585 published studies. Allele frequencies in control population and odds ratios were available for 2,504 and 1,253 risk alleles, respectively. Reported risk allele frequencies were averaged over control populations if the risk allele was identified in more than two studies. However, reported odds ratios were not pooled over different studies and traits even if the risk allele was reported in multiple studies.

To examine common deleterious and neutral SNPs reported in the literature, we used Google Scholar (http://scholar.google.com) and the dbSNP rs number as the search term. The set of tested SNPs was based on 790 deleterious SNPs and 369 neutral nonsynonymous SNPs with an allele frequency of greater than 30% in the HapMap CEU panel. SNPs within known olfactory receptors were excluded. Neutral SNPs were matched to the frequency distribution of deleterious SNPs by acceptance-rejection sampling. As a result, derived allele frequencies are not significantly different between the two sets (Wilcoxon Rank Sum Test, P = 0.79). For each SNP, we searched for reported phenotype associations based on population association or cell-based functional assays. To minimize potential human biases, dbSNP identifiers of deleterious and neutral SNPs were mixed together and Google Scholar search results were manually examined without knowledge of SNP classification. Patents, eQTL associations, conference and poster abstracts, and journals without full-text access were excluded. SNP associations had to be significant after a multiple testing correction. SNP association studies with sample size less than 200 were also not included.

### Genome clustering of deleterious SNPs

To identify genomic regions with exceptionally high rates of deleterious SNPs per coding sequence, 1-Mb sliding windows were scanned across all autosomes with a step size of 0.5 Mb. Assuming that the rate of deleterious SNPs per accessible coding sequence is constant across the genome, a Poisson distribution was used to evaluate the excess number of deleterious SNPs in each window. The expected number of deleterious SNPs per window was set to the product of the genome average (0.51 deleterious SNPs per 1 kb accessible CDS) and the length of accessible coding sequence in the window. Out of 3,549 windows with at least two deleterious SNPs, 70 (2%) with the highest P-value were selected (P<4.5×10^−4^). After excluding regions that were consecutive to or overlapped another region with a smaller P-value, we retained 43 regions.

## Supporting Information

Figure S1 The effect of hitchhiking on neutral and deleterious polymorphism as a function of the rate and strength of advantageous and deleterious mutations.
(PDF)

Figure S2 The ratio of deleterious to neutral SNPs is associated with the rate of recombination.
(PDF)

Figure S3 Hitchhiking regions are enriched for deleterious SNPs.
(PDF)

Table S1 Characteristics of hitchhiking regions.
(XLS)

Table S2 The ratio of deleterious to neutral SNPs in iHS population-specific hitchhiking regions.
(XLS)

Table S3 Ratio of deleterious to neutral SNPs in three population panels for regions identified by multiple tests of selection.
(XLS)

Table S4 Frequency of disease-associated alleles in hitchhiking and non-hitchhiking regions.
(XLS)

Table S5 Disease or phenotype associated SNPs within hitchhiking regions.
(XLS)

Table S6 Genes in hitchhiking regions with deleterious SNPs.
(XLS)

Table S7 Genomic regions enriched for deleterious SNPs.
(XLS)
