# Supplementary material for: Evidence for Hitchhiking of Deleterious Mutations within the Human Genome
Source: PLoS Genet. 2011 Aug 25;7(8):e1002240. doi: 10.1371/journal.pgen.1002240 (PMC3161959; doi:10.1371/journal.pgen.1002240)
Supplement: Figure S1 — The effect of hitchhiking on neutral and deleterious polymorphism as a function of the rate and strength of advantageous and deleterious mutations. (PDF) [file pgen.1002240.s001.pdf]

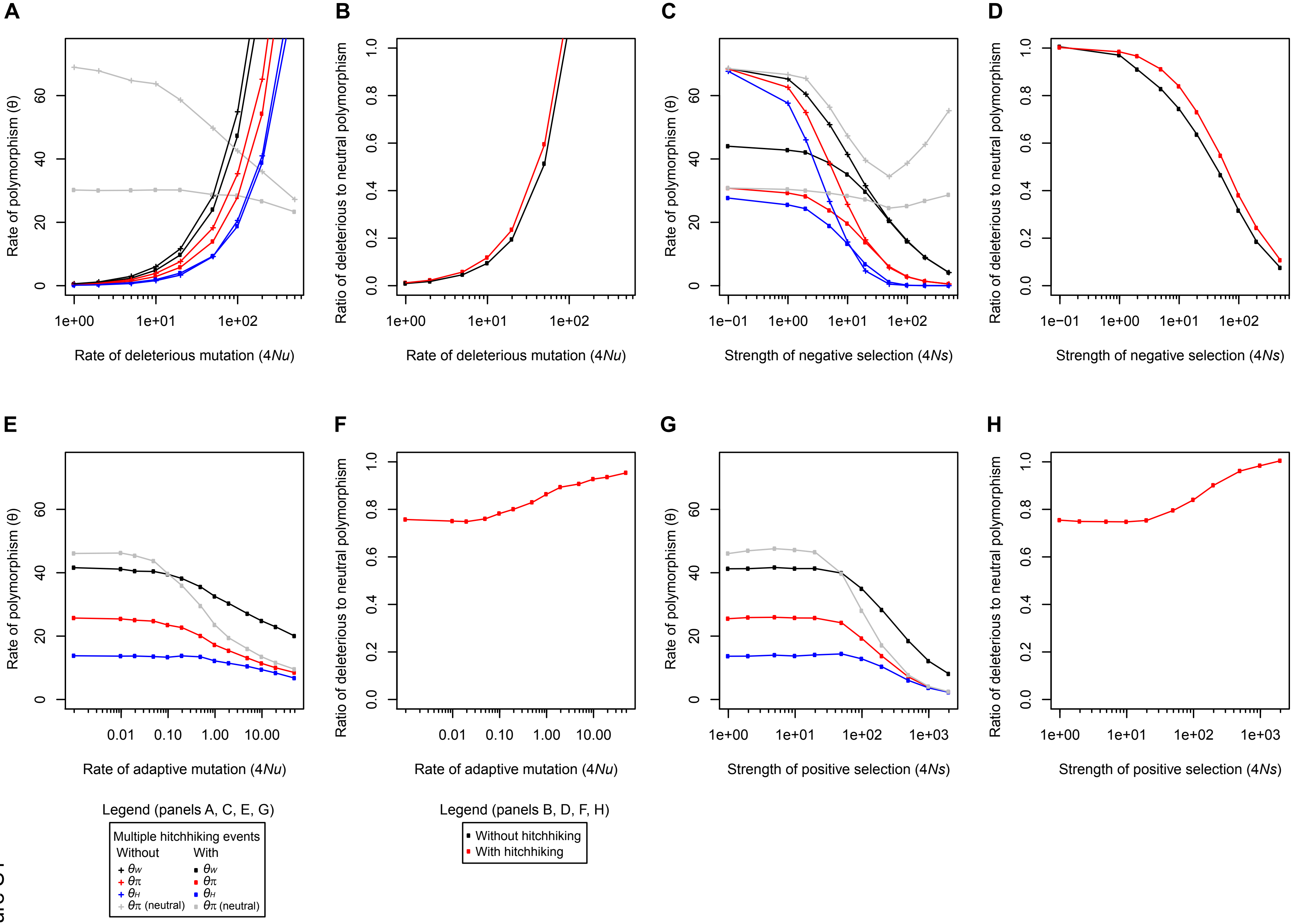
A
B
C
D
E
F
G
H
Rate of polymorphism (θ)
Ratio of deleterious to neutral polymorphism
Rate of deleterious mutation (4Nu)
Strength of negative selection (4Ns)
Rate of adaptive mutation (4Nu)
Strength of positive selection (4Ns)
Legend (panels A, C, E, G)
Multiple hitchhiking events
Without
With
θW
θπ
θH
θπ (neutral)
Legend (panels B, D, F, H)
Without hitchhiking
With hitchhiking

Figure S1

Figure S1. The effect of hitchhiking on neutral and deleterious polymorphism as a function of the rate and strength of advantageous and deleterious mutations. The rate of low, intermediate and high frequency deleterious polymorphism measured by θ_W, θ_π and θ_H, respectively, in the presence (squares) and absence (crosses) of multiple hitchhiking events (A, C, E, G). Average heterozygosity (θ_π) of neutral polymorphism is shown in gray. The ratio of deleterious to neutral polymorphism, measured by θ_W, in the absence (black) and presence (red) of multiple hitchhiking events (B, D, F, H). Unless shown on the x-axis, all panels show the mean of 500 simulations for which $4Nu_n = 70$, $4Nu_d = 70$, $4Ns_d = -10$, $4Nu_a = 0.5$, $4Ns_a = 100$ and $4Nr = 100$, where N is the population size, u is the mutation rate, s is the selection coefficient, and subscripts n, a and d refer to neutral, advantageous and deleterious mutations.
