# Supplementary material for: Evidence for Hitchhiking of Deleterious Mutations within the Human Genome
Source: PLoS Genet. 2011 Aug 25;7(8):e1002240. doi: 10.1371/journal.pgen.1002240 (PMC3161959; doi:10.1371/journal.pgen.1002240)
Supplement: Figure S2 — The ratio of deleterious to neutral SNPs is associated with the rate of recombination. (PDF) [file pgen.1002240.s002.pdf]

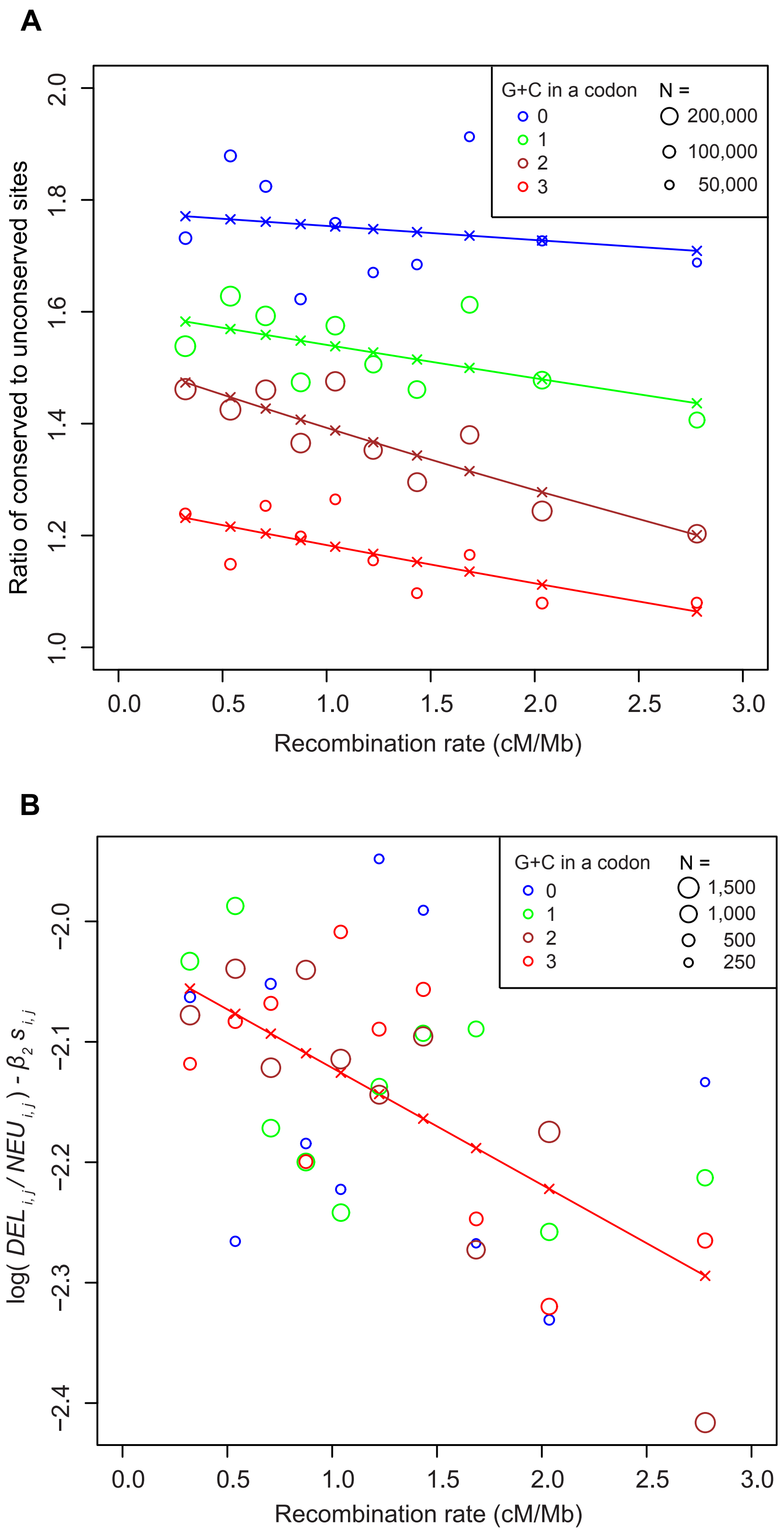

Figure S2. The ratio of deleterious to neutral SNPs is associated with the rate of recombination. After adjusting for the number of potentially deleterious sites (A), the log ratio of deleterious to neutral SNPs is negatively correlated with the rate of recombination for all categories of codons defined by the number of GC nucleotides in them (B). The regression line $\beta_0 + \beta_1 * r_i$ is shown in red. Sample size is indicated by circle size.
