# Supplementary material for: Evidence for Hitchhiking of Deleterious Mutations within the Human Genome
Source: PLoS Genet. 2011 Aug 25;7(8):e1002240. doi: 10.1371/journal.pgen.1002240 (PMC3161959; doi:10.1371/journal.pgen.1002240)
Supplement: Figure S3 — Hitchhiking regions are enriched for deleterious SNPs. (PDF) [file pgen.1002240.s003.pdf]

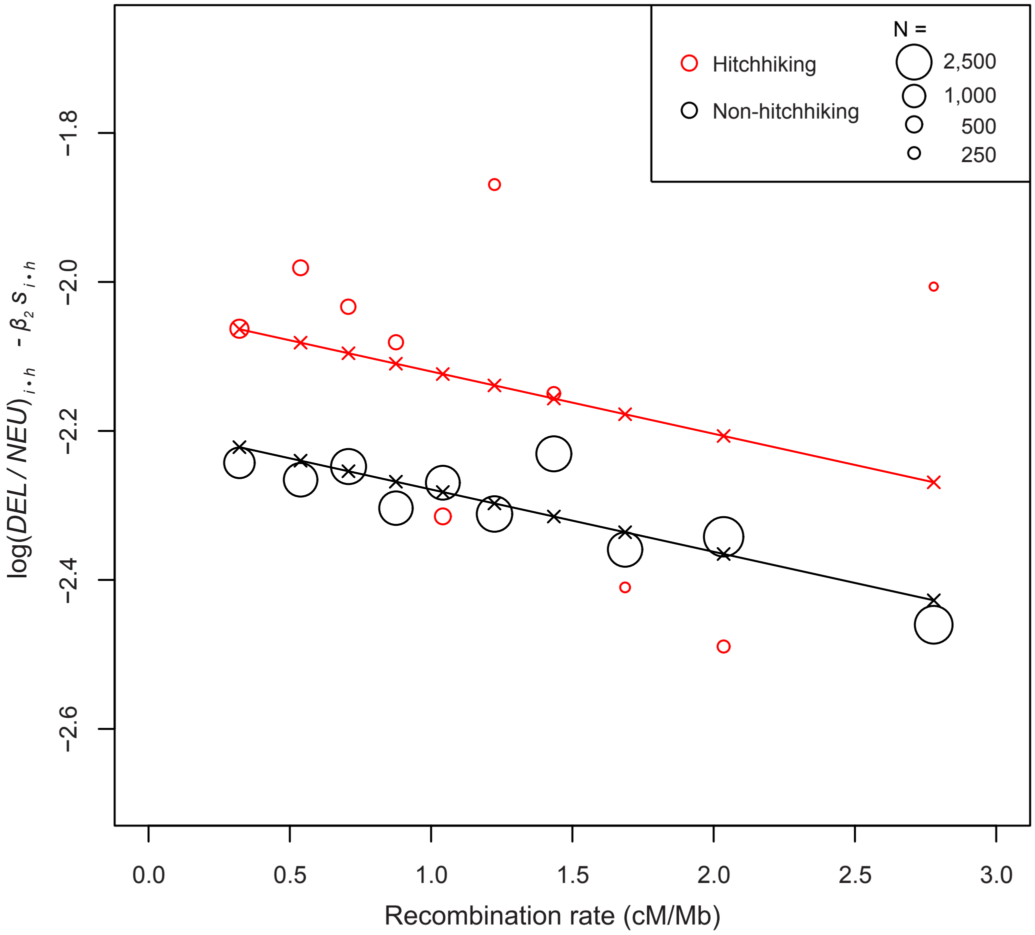

Figure S3. Hitchhiking regions are enriched for deleterious SNPs. The log ratio of deleterious to neutral SNPs (DEL/NEU) after correcting for the number of potentially deleterious sites is significantly higher for hitchhiking (red) compared to non-hitchhiking windows (black). The adjusted log ratio of DEL/NEU was marginalized over the number of GC nucleotides in a codon (j = 0, …, 3) for presentation. The regression line $\beta_0 + \beta_1 * r_i + \beta_3 * h$ is shown for hitchhiking (h = 1) and non-hitchhiking (h = 0) regions. Sample size is indicated by circle size.
